# The Production of Antibody by Invading B Cells Is Required for the Clearance of Rabies Virus from the Central Nervous System

**DOI:** 10.1371/journal.pntd.0000535

**Published:** 2009-10-06

**Authors:** D. Craig Hooper, Timothy W. Phares, Marzena J. Fabis, Anirban Roy

**Affiliations:** 1 Center for Neurovirology, Department of Cancer Biology, Thomas Jefferson University, Philadelphia, Pennsylvania, United States of America; 2 Department of Neurological Surgery, Thomas Jefferson University, Philadelphia, Pennsylvania, United States of America; Centers for Disease Control and Prevention, United States of America

## Abstract

**Background:**

The pathogenesis of rabies is associated with the inability to deliver immune effectors across the blood-brain barrier and to clear virulent rabies virus from CNS tissues. However, the mechanisms that facilitate immune effector entry into CNS tissues are induced by infection with attenuated rabies virus.

**Methodology/Principal Findings:**

Infection of normal mice with attenuated rabies virus but not immunization with killed virus can promote the clearance of pathogenic rabies virus from the CNS. T cell activity in B cell–deficient mice can control the replication of attenuated virus in the CNS, but viral mRNA persists. Low levels of passively administered rabies virus–neutralizing antibody reach infected cells in the cerebellum of B cell–deficient mice but are not sufficient to mediate virus clearance. Production of rabies virus-specific antibody by B cells invading CNS tissues is required for this process, and a substantial proportion of the B cells that accumulate in the CNS of mice infected with attenuated rabies virus produce virus-specific antibodies.

**Conclusions/Significance:**

The mechanisms required for immune effectors to enter rabies virus-infected tissues are induced by infection with attenuated rabies virus but not by infection with pathogenic rabies viruses or immunization with killed virus. T cell activities can inhibit rabies virus replication, but the production of rabies virus–specific antibodies by infiltrating B cells, as opposed to the leakage of circulating antibody across the BBB, is critical to elimination of the virus. These findings suggest that a pathogenic rabies virus infection may be treatable after the virus has reached the CNS tissues, providing that the appropriate immune effectors can be targeted to the infected tissues.

## Author Summary

Every year over 50,000 people die from rabies worldwide, primarily due to the poor availability of rabies vaccine in developing countries. However, even when vaccines are available, human deaths from rabies occur if exposure to the causative virus is not recognized and vaccination is not sought in time. This is because rabies virus immunity induced by the natural infection or current vaccines is generally not effective at removing disease-causing rabies virus from brain tissues. Our studies provide insight into why this is the case and how vaccination can be changed so that the immune response can clear the virus from brain tissues. We show that the type of immune response induced by a live-attenuated rabies virus vaccine may be the key. In animal models, live-attenuated rabies virus vaccines are effective at delivering the immune cells capable of clearing the virus into CNS tissues and promote recovery from a rabies virus infection that has spread to the brain while conventional vaccines based on killed rabies virus do not. The production of rabies-specific antibody by B cells that invade the CNS tissues is important for complete elimination of the virus. We hypothesize that similar mechanisms may promote rabies virus clearance from individuals who are diagnosed after the virus has reached, but not extensively spread, through the CNS.

## Introduction

Rabies viruses spread from peripheral sites of entry to the central nervous system (CNS) tissues via axonal transport thereby bypassing the specialized features of the neurovasculature known as the blood-brain barrier (BBB). Once the virus reaches CNS tissues three alternative outcomes are likely: (1) the BBB remains intact and the infection is lethal due to the absence of an antiviral CNS immune response (2) immune effectors cross the BBB and mediate a CNS antiviral immune response with extensive immunopathology that contributes to the disease, or (3) immune effectors cross the BBB and clear the virus from the CNS without significant pathological consequences. It is well known that in humans naturally infected with rabies virus the latter outcome is exceedingly rare. In addition, CNS inflammation is generally limited in individuals who succumb to rabies [1]. Consequently, it is probable that the BBB remains intact through much of the course of rabies infection in humans. In the absence of a mechanism to compromise the barrier function of the neurovasculature, circulating rabies virus-specific immune effectors, whether raised by the infection or by active or passive immunization, would be unable to mediate an antiviral response in CNS tissues. This may explain why conventional post-exposure treatment of human rabies, consisting of active and passive immunization, is unsuccessful if begun after the appearance of signs of the disease [2]–[4]. At this stage of the infection the virus has likely begun to replicate in the CNS. Thus, the primary function of current post-exposure regimens may be limited to preventing the virus from reaching CNS tissues.

Unlike humans where rabies viruses may take weeks to reach the CNS from the site of exposure [5], the spread of most rabies virus strains to the CNS in mice is rapid with virus generally being detectable in CNS tissues within 48 hours of infection [6]. Nevertheless, normal mice survive infection with laboratory-attenuated strains of rabies virus [7]. While certain of these viruses may be deficient in the capacity to spread from the periphery to the CNS, most of the attenuated rabies virus variants that we have tested spread to and replicate in the CNS but are cleared by immune effectors that cross the BBB and infiltrate neural tissues [7]. In contrast, BBB integrity is maintained and immune effectors do not accumulate in the CNS tissues during infection of mice with common pathogenic rabies virus strains, despite the development of virus-specific immunity in peripheral lymphoid organs and innate immunity in the infected CNS tissues [7],[8]. These observations have led us to speculate that the lethal outcome of infection with wildlife and pathological strains of rabies virus is at least in part due to the evasion of immune clearance as a consequence of the maintenance of BBB integrity [8]. Perhaps the best evidence that this may be the case is that disruption of the BBB in mice infected with a highly lethal silver-haired bat-associated rabies virus (SHBRV), by triggering autoimmune CNS inflammation, promotes the clearance of the virus from the CNS tissues and survival [9]. Due to the associated pathology, the approach of using an autoimmune response to induce elevated BBB permeability and permit rabies virus-specific immune effectors to infiltrate CNS tissues is clearly inappropriate for use in human rabies. On the other hand, the neuroimmune response induced by infection with attenuated rabies virus, which also has the appropriate specificity, is not associated with significant pathology. In this study, we show that the functional changes in the BBB required to deliver immune effectors to the CNS tissues can be induced in mice infected with a lethal rabies virus strain by immunization with a live-attenuated virus vaccine strain but not by administration of killed virus vaccine. Furthermore, our data suggests that clearance of rabies virus from CNS tissues is dependent upon the production of virus-specific antibodies by infiltrating B cells.

## Methods

### Animals, viruses, and antibody treatment

Eight to 12-week old wild-type control 129/SvEv and C57BL6 mice and JHD^−/−^ mice on a C57BL6 background were obtained from the in-house breeding colony at Thomas Jefferson University. RAG-2^−/−^ mice on a 129/SvEv background were obtained from Taconic (Germantown, NY). Mice were infected or immunized intranasally (i.n.) with 10^5^ focus forming units of CVS-F3, CVS-N2c or UV-inactivated CVS-F3 in PBS as previously described [10]. In some experiments, mice were infected and immunized with a combination of the viruses. Where indicated, CVS-F3-infected JHD^−/−^ mice were treated intraperitoneally with 1 mg of the monoclonal, rabies virus glycoprotein-specific, virus-neutralizing antibody 1112 in 500 µl of saline or with the vehicle alone at the time points noted in the figure legends. All procedures were carried out according to the protocols approved by the Institutional Animal Care and Use Committee of Thomas Jefferson University.

### Assessment of blood-brain barrier integrity

BBB integrity was assessed by quantifying the leakage of a low molecular weight fluorescent marker (Na-fluorescein, 376 kDa) from the circulation into CNS tissues as previously described [10]. Briefly, 100 µl of 10% solution of Na-fluorescein was injected intraperitoneally and after 10 minutes mice were anesthetized and cardiac blood was collected followed by transcardial perfusion. Serum samples as well as supernatants of homogenized and centrifuged tissues were clarified by precipitating proteins with 15% TCA and the level of fluorescence measured with a CytoFluor^™^II fluorimeter. The amount of Na-fluorescein in the CNS tissue is normalized to its level in serum by (µg of Na-fluorescein in CNS tissue/mg of tissue)/(µg of Na-fluorescein in serum/µl of serum) and is expressed as a fold increase in fluorescence uptake by comparison with the results obtained from naïve controls.

### Immunohistochemistry

For immunohistochemical analysis, brains from perfused mice were snap frozen in Tissue-Tek O.C.T. Compound (Sakura Finetex, Torrance, CA), sectioned using a Thermo Shandon cryostat (Pittsburgh, PA), and fixed in either 80% acetone or 95% ethanol. Immunoglobulin (Ig) was detected using either biotinylated monoclonal rat anti-mouse kappa light chain (1 hour at 1∶50) (BD Pharmingen, San Jose, CA) followed by Alexa Fluor 568 streptavidin (1 hour at 1∶1000) (Invitrogen, Eugene, OR) or the VECTASTAIN ABC-AP KIT with polyclonal rabbit anti-mouse biotinylated IgG (1∶200) developed using the peroxidase antiperoxidase method and 3′3-diaminobenzidine as substrate (Vector Laboratories, Burlingame, CA) according to the manufacturer's protocol. For the additional staining shown in one of the figure panels (#3D), a 1 hour incubation with 1 mg/ml 1112 was performed prior to detection of Ig. To assess virus infection sections were stained for 1 hour with FITC-conjugated anti-rabies virus nucleoprotein monoclonal antibody (1∶50) (Centocor, Malvern, PA). Photographs were taken with a Nikon digital camera on an Olympus BX-60 microscope.

### Real-time quantitative RT-PCR

Total RNA was isolated from CNS tissue samples and mRNA expression levels of rabies virus nucleoprotein, CD4, CD8, IFN-γ and L13 in CNS tissues were measured by quantitative reverse-transcriptase (RT)-PCR as previously described [10]. Real-time quantitative RT-PCR was carried out on cDNA using specific primer and probe sets and a Bio-Rad iCycler iQ Real Time Detection System (Hercules, CA). The number of copies of specific mRNAs in each sample was determined as previously described [10] and normalized to the mRNA copy number of the housekeeping gene L13 in that sample. Data are expressed as the number of copies of mRNA for a particular gene in a sample per copy of mRNA for the housekeeping gene L13 in that sample.

### Isolation of mononuclear cells, flow cytometry, and ELISPOT analysis

Mononuclear cells were prepared from peripheral blood collected by retro-orbital bleeding in heparinized capillary tubes by centrifugation at 300 g for 20 minutes. The white cell layer was washed in PBS twice before analysis. Mononuclear cells were isolated from CNS tissues as described elsewhere by isolation at the interface of a 30/70 Percoll (Sigma) step gradient centrifuged at 800 g for 25 minutes [11]. For flow cytometry, mononuclear cells were suspended in staining buffer (PBS with 2% FBS and 0.1% NaN_2_) and incubated with anti-CD16/32 (1 ug/10^6^ cells) (2.4G2 BD Pharmingen, San Jose, CA) antibody to prevent non-specific binding. Cells were washed in PBS and incubated with anti-mouse CD19 (1∶1000) (1D3, BD Pharmingen, San Jose, CA) and MHC class-II (1∶1000) (120.1, BD Pharmingen, San Jose, CA) antibodies. Phenotypic characterization of antibody-labeled cells was performed on a BD-FacsCaliber Flowcytometer. CD19-MHC class II double-positive cells were defined as B cells. Numbers of rabies virus-antigen specific antibody secreting B cells were assessed using Millipore Multiscreen HA® ELISPOT plates coated with 5 ug/mL of UV-inactivated whole rabies virus. Peripheral blood or brain derived mononuclear cells were suspended in RPMI media supplemented with 25 mM HEPES and 10% FBS and 200,000 cells were incubated in each well for 18 hours. Plates were washed and bound rabies virus-specific antibodies were detected by addition of alkaline-phosphatase conjugated anti-mouse IgG antibody (1∶500) (Sigma, St. Louis, MO) followed by BCIP/NBT substrate. Spots were counted using a dissecting microscope.

### Statistical analyses

Results are expressed as the mean±standard error mean (S.E.M.). Statistical significance of the differences between groups was tested using the Mann-Whitney test and the symbol * indicates a p value<0.05.

## Results

### Lethal rabies virus infection can be prevented by immunization with live-attenuated, but not killed rabies virus

Mouse models are not considered to be particularly suited to studies of post-exposure prophylaxis (PEP) with rabies due to the rapid spread of the viruses to the CNS. However, our prior studies suggest that the lethal outcome of rabies in mice is more a consequence of the inability to deliver immune effectors into CNS tissues than its spread [8]. In our view, the key feature is that BBB integrity is maintained during infection with lethal rabies viruses, while infection with attenuated rabies virus variants causes enhanced BBB permeability and a virus-clearing CNS immune response [7],[10]. The reason for this difference could be that infection with highly pathological rabies virus strains causes the inhibition of immune mechanisms that mediate the changes in BBB function necessary for rabies-specific immune effectors to cross. Alternatively, these may not be triggered due to a subtle difference in rabies virus immune mechanisms induced by pathogenic and attenuated viruses. To distinguish between these two possibilities, mice were infected with the attenuated CVS-F3 variant or immunized with killed CVS-F3 and then 3 or 5 days later were super-infected with the pathogenic CVS-N2c rabies virus. CVS-F3 was administered first because the virus spreads to, and replicates in the CNS more slowly than CVS-N2c (data not shown). The delays were limited to 3 and 5 days so that the CVS-N2c infection would have the 48 hours required to spread to the CNS before the appearance of serum rabies virus-specific antibodies approximately 8 days following CVS-F3 infection [10]. To control for unanticipated effects caused by the administration of either immunogen, groups of mice were also given both live and inactivated CVS-F3. As shown in Table 1, administration of an inactivated CVS-F3 vaccine preparation that is effective when given several weeks before a CVS-N2c challenge does not protect when given 3 or 5 days prior to challenge. On the other hand, the majority of mice infected with CVS-F3 as recently as 3 days previously survive CVS-N2c infection regardless of whether or not inactivated virus is also administered. These results suggest that the processes required to clear pathogenic rabies virus from CNS tissues are induced by infection but not immunization with CVS-F3.

**Table 1:** Survival of mice immunized or infected with CVS-F3 prior to infection with CVS-N2c.

Initial Infection or Immunization	Secondary Infection (Delay in Days)	Survival (Percent)
None	CVS-N2c	0/5 (0)
CVS-F3, live	none	5/5 (100)
CVS-F3, live	CVS-N2 c (3)	4/5 (80)
CVS-F3, live	CVS-N2c (5)	5/5 (100)
CVS-F3, inactivated	CVS-N2c (3)	0/5 (0)
CVS-F3, inactivated	CVS-N2c (5)	0/5 (0)
CVS-F3, inactivated	CVS-N2c (21)	10/10 (100)
CVS-F3, live and inactivated	CVS-N2c (3)	3/5 (60)
CVS-F3, live and inactivated	CVS-N2c (5)	5/5 (100)

### T cell activities promote long-term survival in CVS-F3-infected mice but cannot clear the virus

**Figure 1:**
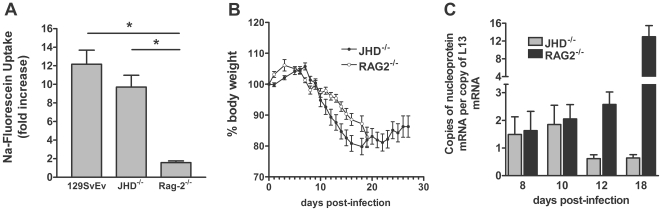
The T cell response to CVS-F3 infection in the absence of B cells mediates enhanced BBB permeability and long-term survival. The extent of BBB permeability in the cerebellum of wild-type, JHD^−/−^ and RAG-2^−/−^ mice, infected i.n. with CVS-F3 10 days previously, is shown in panel A. Weight loss over the course of infection in JHD^−/−^ and RAG-2^−/−^ mice is shown in panel B, while the number of copies of rabies virus nucleoprotein mRNA at different times of infection in these animals is presented in panel C. BBB permeability changes are shown as the mean±S.E.M. fold increase in Na-Fluorescein uptake in the tissues with the levels from uninfected mice taken as 1. Weight is expressed as the mean±S.E.M. percent body weight with the weight on day 0 being taken as 100% and copies of nucleoprotein mRNA in the tissues are expressed per copy of L13 mRNA in the same sample.

**Figure 2:**
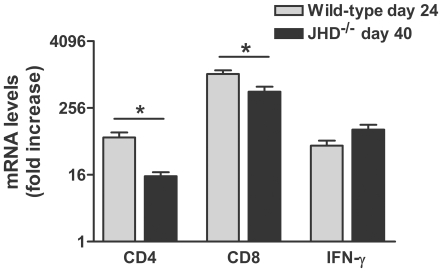
Prolonged IFN-γ mRNA expression in the CNS tissues of CVS-F3-infected JHD^−/−^ mice. Wild-type and JHD^−/−^ mice were infected i.n., with CVS-F3 and euthanized at 24 or 40 days following infection to assess CD4, CD8 and IFN-γ mRNA levels using quantitative RT-PCR. Data is expressed as the mean±S.E.M. fold increase with the levels in tissues from uninfected mice taken as 1.

JHD^−/−^ mice lack B cells but have functional T cells and, unlike RAG-2^−/−^ mice, which lack both T and B cells, are capable of elevating fluid-phase BBB permeability in response to the infection (Fig. 1A). These mice are therefore suitable for analyzing the effects of antibody administration on CVS-F3 infection. As a preface to such studies we compared the course of CVS-F3 infection in JHD^−/−^ and RAG-2^−/−^ mice. As is the case for wild-type mice infected with CVS-F3 [10], both JHD^−/−^ and RAG-2^−/−^ mice lose weight as the infection progresses (Fig. 1A). However, while RAG-2^−/−^ mice continue to lose weight and die approximately 20 days following infection, up to 70% of JHD^−/−^ mice survive past this time-point, most showing a modest weight gain (Fig. 1B). At the same time virus replication, which continues to increase in RAG-2^−/−^ mice, becomes reduced in the JHD^−/−^ mice (Fig. 1C). These JHD^−/−^ mice exhibit signs of rabies infection including ataxia and partial paralysis but survive the infection for extended periods of time ( >40 days). This raises the possibility that T cell activities may be able to partly control the virus infection independently of antibody. To gain insight into the contributing T cell subsets, we compared the levels of CD4, CD8 and IFN-γ mRNAs in CNS tissues from wild-type conventional mice 24 days after CVS-F3 infection when there is little virus replication remaining (see below) and JHD^−/−^ mice 40 days after infection. As can be seen in Fig. 2, the levels of CD4 and CD8 mRNA are somewhat lower in the JHD^−/−^ mice but IFN-γ mRNA levels have remained relatively high and may therefore be contributing to the control of virus replication.

### Leakage of antibody across the BBB may not be sufficient to clear CVS-F3 from CNS tissues

**Figure 3:**
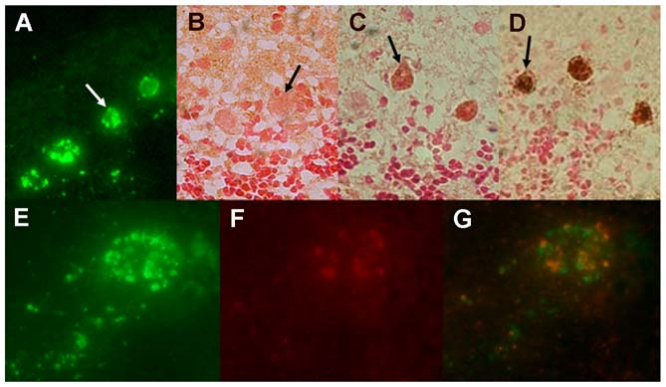
Rabies virus specific antibodies administered to CVS-F3-infected JHD^−/−^ mice have limited access to infected CNS tissues. At 7 and 9 days post-infection, CVS-F3-infected JHD ^−/−^ mice were treated with 1 mg of the rabies glycoprotein-specific, virus-neutralizing mouse monoclonal antibody 1112 (A, C, D, E, F, G) or saline vehicle alone (B). Several hours after the second treatment mice were transcardially perfused, CNS tissues removed and sections from the cerebellum were stained for rabies virus infection with FITC-anti-nucleoprotein monoclonal antibody (panels A and E; green), polyclonal rabbit anti-mouse biotinylated IgG (panels B and C; brown), additional 1112 antibody followed by the polyclonal rabbit anti-mouse biotinylated IgG (panel D; brown), or a combination of FITC-anti-nucleoprotein monoclonal antibody and rhodamine-conjugated anti-mouse Ig. Arrows in panels A-D identify Purkinje cells, a cell population that is extensively infected. Panels E-G show close-ups of a single cell stained with FITC-anti-nucleoprotein monoclonal antibody and rhodamine-conjugated anti-mouse Ig photographed using filters for FITC (panel E), rhodamine (panel F) and a combination red/green filter that shows both stains simultaneously (panel G).

The inability of JHD^−/−^ mice to clear CVS-F3 from the CNS reaffirms the importance of rabies virus-specific antibodies in this process. However, little is known with respect to how these antibodies may be delivered to infected CNS tissues. Our studies of mice clearing CVS-F3 suggest that the leakage of naturally developing antibodies from the circulation into the CNS tissues may be minimal since elevated BBB permeability occurs before serum antibody titers peak [10]. In addition, over the short term, extensive fluid phase exchange across the BBB is seen but little accumulation of markers of the molecular mass of antibody is detectable [12]. Nevertheless, it may be expected that some antibody would cross the BBB in conjunction with infiltrating immune cells and, over time, sufficient levels may accumulate to impact virus replication. To examine this possibility, JHD^−/−^ mice were treated with 1 mg of the mouse IgG1 monoclonal rabies virus-neutralizing, glycoprotein-specific antibody 1112, which is highly effective in post-exposure treatment models [13], on each of days 7 and 9 post-infection when BBB permeability is at a peak. Several hours later, CNS tissues were obtained and stained with antibodies specific for rabies nucleoprotein and for mouse IgG to determine if there was any antibody associated with infected cells. While extensive infection of Purkinje cells can be readily detected with nucleoprotein-specific antibodies in sections from the cerebellum of JHD^−/−^ mice (Fig. 3A), as expected, there is no evidence of IgG in sections from animals that had not received antibody (Fig. 3B). IgG-specific staining of Purkinje cells in sections from mice receiving 1112 antibody could be detected (Fig. 3C) but the treatment of these sections with additional 1112 antibody *in vitro* prior to IgG detection resulted in more extensive staining (Fig. 3D). When cells stained for both nucleoprotein (Fig. 3E,G green) and antibody (Fig. 3F,G red) were examined more closely, distinct inclusions of nucleoprotein and antibody/glycoprotein can be seen. These findings suggest that low levels of 1112 antibody can leak from the circulation to interact with rabies virus-infected cells in the CNS tissues provided that their application coincides with elevated BBB permeability.

**Figure 4:**
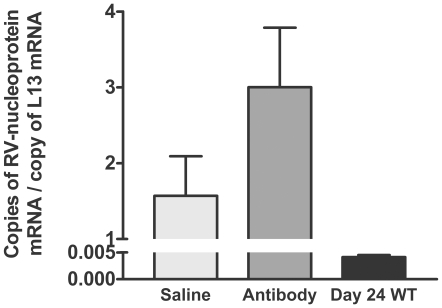
The administration of rabies virus neutralizing antibodies fails to clear CVS-F3 from the CNS of JHD^−/−^ mice. JHD ^−/−^ mice were infected i.n. with CVS-F3 and treated at 7, 9, 11, 13, and 15 days post-infection with saline vehicle alone or the rabies-specific antibody 1112. Three weeks after the final treatment mice were transcardially perfused, cerebellar tissues were removed, and virus replication was estimated by quantifying nucleoprotein mRNA levels. Virus replication was similarly estimated in cerebellar tissues from wild-type mice infected with CVS-F3 24 days previously. Virus replication is expressed as the mean±S.E.M. copies of rabies virus nucleoprotein mRNA per copy of the housekeeping gene L13 mRNA in the tissue sample.

To determine whether 1112 antibody administration to CVS-F3-infected JHD^−/−^ mice leads to the clearance of the virus from CNS tissues, we administered saline or 1 mg of the antibody 5 times at two day intervals between days 7 and 15 post-infection. This antibody dose regimen achieved a half-maximal serum rabies-specific antibody titer of approximately 1/240, which is roughly equivalent to the serum titer found in normal mice 8 days post-infection with CVS-F3, during the period of time when BBB permeability is maximal. Viral nucleoprotein mRNA levels in the CNS tissues of surviving animals, both saline and antibody treated, were substantial several weeks later (Fig. 4) at a time when they are virtually undetectable in wild-type mice [10]. Moreover, no impact on the health or survival of the mice was noted.

### Clearance of rabies virus from CNS tissues is dependent upon antibody production by infiltrating B cells

**Figure 5:**
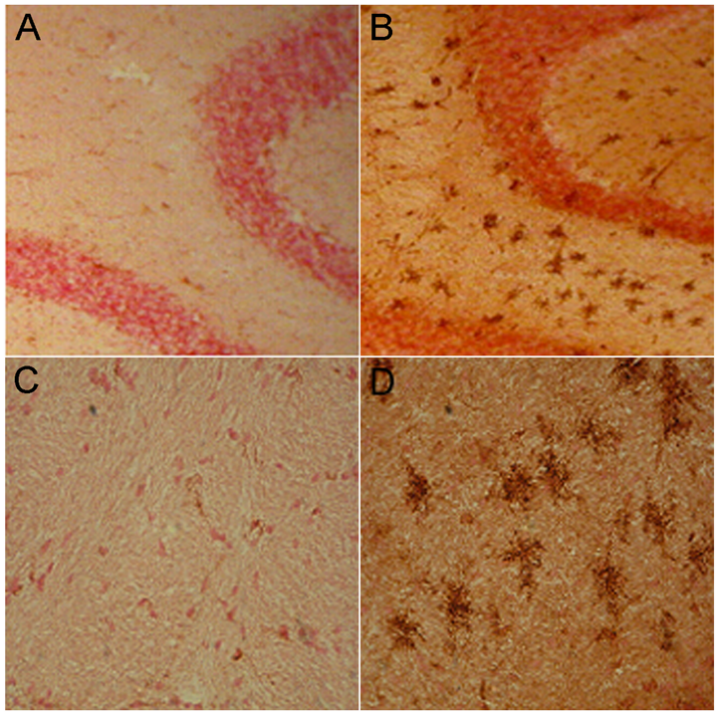
Pattern of antibody appearance in the CNS tissues of CVS-F3-infected mice. Sections from the cerebella of wild-type mice either uninfected (A and C) or infected with CVS-F3 12 days previously (B and D) were stained for IgG (brown). Photomicrographs taken at low magnification are shown in panels A and B and at higher magnification in C and D.

**Figure 6:**
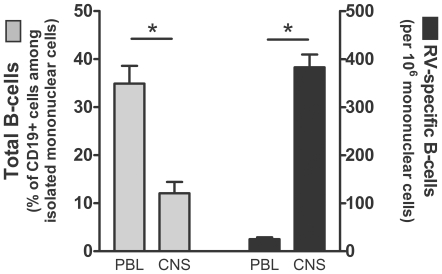
Rabies virus-specific antibody secreting B cells accumulate in the CVS-F3-infected CNS. Mononuclear cells isolated from peripheral blood lymphocytes (PBL) and brain (CNS) were assessed for surface phenotype using flow cytometry and for the numbers of rabies virus-specific antibody secreting cells by ELISPOT. The percentage of B cells, identified as positive for both CD19 and MHC class II, among total mononuclear cells is presented on the left side of the figure while the proportion of the total mononuclear cells producing rabies virus-specific antibodies is presented on the right side of the figure.

CVS-F3 clearance from the CNS tissues of wild-type mice occurs prior to the development of high titers of circulating virus-neutralizing antibodies (VNA) and after BBB permeability has peaked [10]. However, B cells that have infiltrated the CNS tissues express high levels of κ-light chain mRNA during this time period indicating that there is likely to be substantial antibody production in the CNS tissues [10]. To assess this possibility more directly, we used antibodies specific for mouse IgG to stain CNS tissues from wild-type mice infected 12 days previously with CVS-F3. Extensive foci of antibody are seen throughout the cerebellum (Fig. 5). At higher magnification the antibodies appear to be diffusing in stellate patterns from the foci (Fig. 5). To determine if B cells may be the source of these antibodies and whether or not they are likely to be rabies virus-specific, we assessed rabies virus-specific antibody production by B cells from the peripheral blood and CNS tissues of CVS-F3-infected mice. While the proportion of CD19^+^ B cells in mononuclear cells recovered from the CNS tissues of CVS-F3-infected mice is lower than in peripheral blood from the same animals, the fraction of the cells that produce rabies virus-specific antibodies is considerably higher (Fig. 6). This suggests that B cells producing rabies virus-specific antibodies either selectively invade or expand in the CNS tissues in response to CVS-F3 infection.

## Discussion

Prompt administration of PEP is the recommended course for an individual who has come in contact with a rabid animal. Since this prevents the development of clinical rabies it is impossible to be certain how many of the tens of thousands of people who receive PEP on an annual basis have actually been infected with the virus. It is also impossible to know how far the rabies virus may have spread before being cleared by the immune effectors provided or induced by PEP and the infection. The commonly held view that pathogenic wildlife rabies virus that has spread to the CNS cannot be cleared by immune mechanisms is supported by the absence of significant immune cell infiltration into the CNS tissues of individuals who die from rabies [1] and the failure of PEP in individuals that have developed signs of rabies [2],[4]. Our studies in animal models of rabies suggest that this is a consequence of the inability of virus-specific immune effectors to cross the BBB and enter CNS tissues infected with pathogenic rabies viruses [7],[8]. The rabies virus-specific immune effectors that are raised in lethally infected mice are able to clear rabies virus from the CNS if provided access across the BBB. For instance, when the BBB is compromised by the induction of autoimmune CNS inflammation, rabies-specific immune effectors infiltrate CNS tissues and can clear the highly pathogenic SHRBV [9]. In addition, the adoptive transfer of immune effectors recovered from mice lethally infected with SHBRV results in clearance of the attenuated CVS-F3 virus from the CNS tissues of mice lacking T and B lymphocytes [8]. In contrast, the transfer of cells from mice clearing CVS-F3 has no impact on the outcome of SHBRV infection [8]. Regardless of the infecting virus strain, elements of the innate immune response that are important for the early control of virus replication and for attracting immune cells into infected tissues are induced [7],[8],[10]. These findings led us to speculate that functional changes at the BBB required to provide immune effectors access to the CNS tissues are induced during infection with attenuated rabies virus strains but not during pathological rabies virus infection [7]–[9]. A key issue examined in this study is whether or not this is due to an inhibitory process triggered by infection with pathogenic rabies virus. If so, it may be expected that BBB integrity would be maintained during infection with both pathogenic and attenuated rabies viruses and the outcome would be lethal, but it is not. Infection with an attenuated rabies virus induces BBB integrity changes and immune effector entry into CNS tissues regardless of whether or not there is also an ongoing infection with pathogenic rabies virus. However, protection is not provided by immunization with killed virus. We therefore conclude that the generation of a rabies virus-specific immune response in the periphery is not sufficient to clear pathogenic rabies viruses from the CNS tissues. A mechanism selectively induced by infection with attenuated rabies virus, likely manifested at the BBB, is necessary to provide immune effectors access to CNS tissues.

To gain further insight into the mechanism of rabies virus clearance from the CNS tissues, we have used gene-deleted mice to study the role(s) of different antiviral immune effectors in the CNS tissues of mice clearing the attenuated rabies virus CVS-F3. Mice lacking T and B cells cannot clear this virus and die from the infection [8],[14]. CD8 T cells contribute to, but are not required for the clearance of CVS-F3 as clearance is merely delayed in mice without this cell population [14],[15]. On the other hand, JHD^−/−^ mice, which lack B cells but have functional CD4 and CD8 T cells, often survive CVS-F3 infection over extended periods despite being unable to clear virus from CNS tissues and exhibiting neurological symptoms. This leads us to conclude that elements of the T cell response, likely including IFN-γ production by CD4 and CD8 T cells, can control certain features of the infection that make significant contributions to its lethality but that antibody is required for virus clearance. To examine the contribution of circulating antibody to virus clearance from CNS tissues, we administered high levels of the rabies virus neutralizing mouse monoclonal 1112 antibody to CVS-F3-infected JHD^−/−^ mice during the stage of infection when BBB permeability is maximal. While leakage of a 150 kDa molecular weight marker from the circulation into the CNS tissues of CVS-F3-infected mice is minimal over a 4-hour period [12], antibodies present in the circulation over a more extensive period of time can evidently leak into the CNS tissues of the infected mice. 1112 antibody was found associated with the Purkinje cells in the cerebellum that express high levels of rabies virus antigen. The antibody was primarily localized in inclusion bodies which is consistent with previous in vitro studies showing that 1112 antibody is rapidly internalized by rabies virus-infected neuroblastoma cells where it accumulates in intracellular vesicles [13]. Of note in our studies is that the intracellular inclusions of glycoprotein-specific 1112 are generally distinct from inclusions of the virus nucleoprotein. The amounts of antibody reaching rabies virus-infected cells *in vivo* appears to be relatively low as considerably greater amounts of the antibody can bind to the cells when applied to tissue sections *in vitro*. While it is possible that even low levels of virus-neutralizing antibody may impact the replication and spread of the virus while BBB permeability is enhanced, treatment of CVS-F3-infected JHD^−/−^ mice with 1112 antibody failed to clear the virus.

It should be noted with respect to the origin of the antibodies that participate in rabies virus clearance that serum rabies virus-specific antibody titers peak some time after BBB integrity has been restored [10]. The presence of cells expressing the B cell phenotypic marker CD19 and mRNAs specific for κ- light chain in the CNS tissues of mice clearing CVS-F3 [8],[10] led us to examine the possibility that rabies virus-specific antibodies are produced by infiltrating B cells. The current findings indicate that this is the case. Focal concentrations of antibodies can be readily detected in the CNS tissues of mice clearing CVS-F3 and a high proportion of B cells recovered from the tissues produce rabies virus-specific antibodies *in vitro*. This leads us to conclude that the high levels of antibodies required for rabies virus clearance from the CNS tissues are produced at the site of infection rather than diffusing in from the circulation. In this case, passively administered antibody during PEP would primarily impact virus in the periphery and an active immune response leading to elevated BBB permeability and immune effector delivery to the CNS tissues would likely be required to clear virus from the CNS.

As certain of the aspects of BBB function required for immune cell infiltration are unchanged by CNS infection with pathogenic rabies viruses [8], once the virus has reached the CNS a PEP protocol capable of altering the BBB, so that virus-specific immune effectors can reach the infected tissues, is required. Inactivated CVS-F3 can induce rabies virus-specific T and B cells, but fails to promote recovery from CVS-N2c infection over a time frame during which the administration of live CVS-F3 is therapeutic. We consider that this is a consequence of the inability of the inactivated virus to induce the functional changes in the BBB that are required for antiviral immune effectors to enter CNS tissues. In our view, administration of a live-attenuated rabies virus vaccine is the most reasonable, currently available, approach to providing the appropriate immune effectors access to the CNS tissues. The results of our experiments with a new, highly attenuated recombinant rabies virus vaccine which expresses three copies of a mutated glycoprotein gene, strongly support this hypothesis [16]. In these studies, the triple G vaccine was shown to promote immune effector delivery into CNS tissues and normal mice were found to survive the intracranial injection of a mixture of the vaccine virus and a highly pathogenic dog strain which was nearly 100% lethal when administered alone [16]. The triple G vaccine also proved effective in the post-exposure treatment of mice infected with a highly pathogenic dog rabies virus several days previously [16]. However, when UV-inactivated and given peripherally to mimic conventional post-exposure vaccination, there was little protective effect [16]. In addition to boosting the antiviral response, attenuated rabies virus vaccines spread to the CNS where they trigger the mechanisms required for T cells and B cells to enter the tissues and clear, not only the attenuated, but also pathogenic rabies viruses. It is clear from the commonly lethal outcome of rabies that these mechanisms are not induced in a timely fashion in the context of the spread of a wildlife rabies virus to the human CNS.
